# Modifiable risk factors for multiple sclerosis have consistent directions of effect across diverse ethnic backgrounds: a nested case–control study in an English population-based cohort

**DOI:** 10.1007/s00415-023-11971-0

**Published:** 2023-09-07

**Authors:** Benjamin M. Jacobs, Pooja Tank, Jonathan P. Bestwick, Alastair J. Noyce, Charles R. Marshall, Rohini Mathur, Gavin Giovannoni, Ruth Dobson

**Affiliations:** 1 Centre for Preventive Neurology, Wolfson Institute of Population Health, Queen Mary University London, London, EC1M 6BQ UK; 2 Department of Neurology, Royal London Hospital, London, UK; 3 Centre for Primary Care, Wolfson Institute of Population Health, Queen Mary University London, London, UK; 4 Blizard Institute, Queen Mary University London, London, UK

**Keywords:** Multiple sclerosis, Epidemiology, Risk factors, Ethnicity, CPRD

## Abstract

**Background:**

Multiple sclerosis is a leading cause of non-traumatic neurological disability among young adults worldwide. Prior studies have identified modifiable risk factors for multiple sclerosis in cohorts of White ethnicity, such as infectious mononucleosis, smoking, and obesity during adolescence/early adulthood. It is unknown whether modifiable exposures for multiple sclerosis have a consistent impact on risk across ethnic groups.

**Aim:**

To determine whether modifiable risk factors for multiple sclerosis have similar effects across diverse ethnic backgrounds.

**Methods:**

We conducted a nested case–control study using data from the UK Clinical Practice Research Datalink. Multiple sclerosis cases diagnosed from 2001 until 2022 were identified from electronic healthcare records and matched to unaffected controls based on year of birth. We used stratified logistic regression models and formal statistical interaction tests to determine whether the effect of modifiable risk factors for multiple sclerosis differed by ethnicity.

**Results:**

We included 9662 multiple sclerosis cases and 118,914 age-matched controls. The cohort was ethnically diverse (MS: 277 South Asian [2.9%], 251 Black [2.6%]; Controls: 5043 South Asian [5.7%], 4019 Black [4.5%]). The age at MS diagnosis was earlier in the Black (40.5 [SD 10.9]) and Asian (37.2 [SD 10.0]) groups compared with White cohort (46.1 [SD 12.2]). There was a female predominance in all ethnic groups; however, the relative proportion of males was higher in the South Asian population (proportion of women 60.3% vs 71% [White] and 75.7% [Black]). Established modifiable risk factors for multiple sclerosis—smoking, obesity, infectious mononucleosis, low vitamin D, and head injury—were consistently associated with multiple sclerosis in the Black and South Asian cohorts. The magnitude and direction of these effects were broadly similar across all ethnic groups examined. There was no evidence of statistical interaction between ethnicity and any tested exposure, and no evidence to suggest that differences in area-level deprivation modifies these risk factor-disease associations. These findings were robust to a range of sensitivity analyses.

**Conclusions and relevance:**

Established modifiable risk factors for multiple sclerosis are applicable across diverse ethnic backgrounds. Efforts to reduce the population incidence of multiple sclerosis by tackling these risk factors need to be inclusive of people from diverse ethnicities.

## Introduction

Multiple sclerosis (MS) is an autoimmune disorder of the central nervous system (CNS) affecting over 2.2 million people worldwide [1]. Despite MS being diagnosed in people from all ethnic and ancestral backgrounds [2, 3], most observational studies of MS risk have focussed on individuals from White ethnic backgrounds [4–6]. Contemporary studies of MS risk across different ethnic groups in high-income countries suggest a similar incidence in persons of Black and White ethnicity, with lower incidence in persons of South Asian and East Asian ethnicity [2, 3, 7].

MS susceptibility is influenced by both genetic factors [8–11] and exposure to potentially modifiable triggers, including infectious mononucleosis (IM), obesity during adolescence/early adulthood, vitamin D deficiency, and cigarette smoking [4, 5]. To date, the association between exposure to environmental and/or lifestyle factors and MS has been explored through observational studies and reinforced through Mendelian randomisation (MR) [12–17]. However, the overwhelming majority of these studies have focussed on populations of predominantly White ethnicity; efforts to examine modifiable exposures and subsequent MS risk in diverse ethnic groups have been conducted on a smaller scale [3, 18, 19].

It remains unclear whether established exposures associated with MS risk have the same effect across diverse ethnic and racial groups [3]. MS is a heterogeneous disease in terms of presentation, clinical course, and response to treatment [20]. There is a body of evidence showing variation in age of onset, first symptoms, mortality, disease activity, and progression between individuals from different ethnic and racial backgrounds [3, 22–36]. The observed heterogeneity between different ethnic groups may be a result of either genetic or modifiable drivers of disease severity [21]. Studying the underlying causes of this heterogeneity will help to disentangle biological drivers (such as genetic heterogeneity or differential influence of risk factors) from non-biological drivers, such as systemic racism and unequal access to healthcare.

The Clinical Practice Research Datalink (CPRD) is a population-based data resource in the United Kingdom (UK). CPRD collates pseudonymised, routinely recorded electronic health record data from primary care practices across the UK, encompassing a variety of clinical observations, measurements, diagnostic codes, tests, and other healthcare encounters. All data are anonymised, and CPRD performs checks to ensure the data are of high quality and accuracy [37]. Through linkage to secondary care datasets (such as Hospital Episode Statistics; HES) and Office for National Statistics data (ONS, such as area-level deprivation data), CPRD can be used to explore a wide range of associations between exposures and health-related outcomes [37]. In total, CPRD covers >10% of the UK population, and therefore provides statistical power to study diseases such as MS with relatively low prevalence (0.2–0.5% in the UK) [37–44].

In this study, we use data from CPRD to determine whether modifiable risk factors for MS previously reported in predominantly White cohorts are of similar relevance for persons of South Asian and Black ethnic backgrounds.

## Methods

### Cohort and data sources

Data for this study were obtained from CPRD Aurum linked to three HES datasets: Outpatients (OP), Admitted Patient Care (APC), and Accident and Emergency (AE), relating to outpatient, inpatient, and emergency care encounters, respectively. These data relate to hospitals in England only, i.e. they do not include Scotland, Wales, or Northern Ireland. HES-OP data have been collected since 2003–2004; the linked HES-OP dataset used in this study covered the period April 2003 to October 2020. Set 22 of HES-APC data covering the period 1997–March 2021 inclusive were used. HES-AE data were collected from 2007, and set 21 of the HES-AE data which covers April 2007 to March 2020 inclusive were used.

We used linked geographical data to infer the deprivation status and urban/rural location of participants. CPRD links individual patient postcodes and GP practice IDs to the UK census geography using lower layer super output areas (LSOA), comprising an average of ~ 1600 individuals per LSOA. The index of multiple deprivation (IMD) is a composite area-level metric of deprivation calculated as a weighted combination of various factors (such as employment, education, and income). We used the 2019 patient-level update to the IMD, which is only available for participants in England. We also obtained the rural/urban classification for each GP practice postcode determined by the Office for National Statistics based on the 2011 census.

### Participants (definitions of cases and controls)

Data were extracted from the May 2022 build of CPRD Aurum. In total, 41,092,910 patients had data with sufficient quality for inclusion (i.e. >  = 1 day of follow-up between 01/01/1990 and 01/03/2022, and recorded gender).

**Fig. 1:**
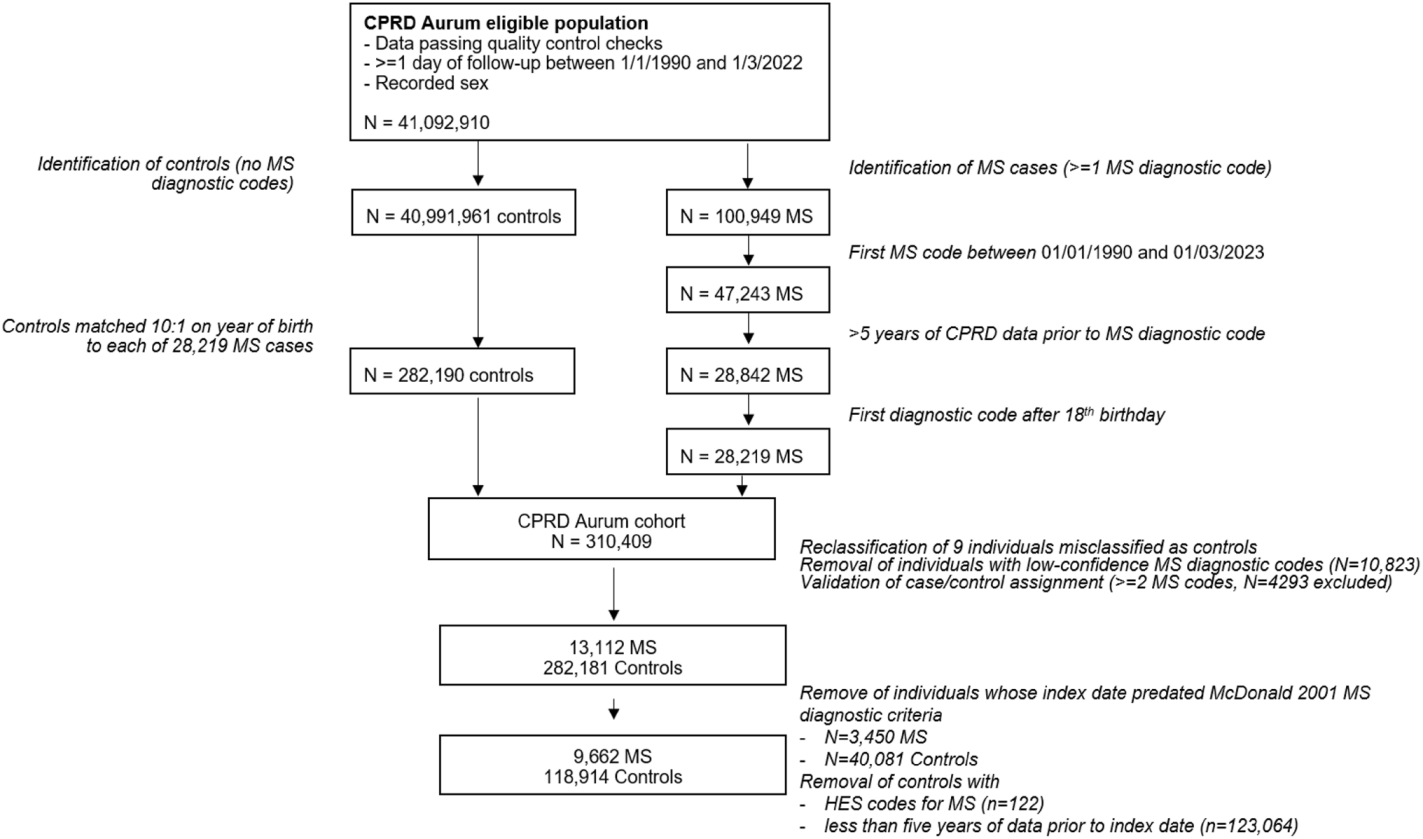
Selection of cases and controls from the CPRD Aurum primary care dataset. The numbers in the boxes indicate the number of participants remaining after application of each inclusion/exclusion criterion. Note that the requirement for a single diagnostic MS code was applied by CPRD to assign case/control status, but we subsequently restricted the case cohort to those with two or more diagnostic MS codes

Multiple sclerosis (MS) cases were defined based on the following criteria (Fig. 1):

- Potential MS cases were identified by CPRD using a lenient case definition of  > = 1 MS diagnostic code in the primary care electronic health records;
- We then validated MS cases using a more stringent definition, stipulating the presence of  > = 2 recorded MS diagnostic codes in the primary care electronic health records;
- Earliest MS diagnostic code recorded at age 18 or later;
- Earliest MS diagnostic code recorded after 1 January 2001 (the year of the initial McDonald criteria for standardising MS diagnoses [45]);
-  >  = 5 years of continuous CPRD data prior to earliest MS diagnostic code;
- Eligible for linkage to external data sources (Hospital episode statistics and/or practice-level indices of multiple deprivation data).

To improve the accuracy of the case/control definition, we excluded participants with only one diagnostic code highly suggestive of MS, and those with diagnostic codes suggestive of other inflammatory/demyelinating conditions. The date of the earliest MS diagnostic code was used as a proxy for date of diagnosis. From an initial cohort of 310,409 people provided by CPRD, we identified 28,228 possible cases with either MS or neuroinflammatory disease codes and 282,181 controls—of note, we found nine individuals classified as controls by CPRD who had an MS diagnostic code in their records. We then excluded 10,823 people with nonspecific codes and codes for other inflammatory disorders, retaining the 17,405 people with > = 1 ‘definite’ MS diagnostic code in the primary care data. Of these 17,405 cases, we excluded 4,293 with a single diagnostic code, leaving 13,112 cases. We excluded a further 3,450 MS cases with their first recorded diagnostic code prior to the advent of the 2001 McDonald criteria for diagnosis, resulting in 9,662 cases.

Controls were defined as all individuals with sufficient quality data without any MS diagnostic codes in their records (*n* = 40,991,961). For each MS case, controls were matched in a 10:1 ratio on year of birth (Fig. 1). Each control was assigned an index date corresponding to the date of MS diagnostic code report for their matched case. Controls were excluded if they had less than 5 years of antecedent continuous CPRD registration data prior to the index date of their matched case, if their index date occurred prior to the publication of the 2001 McDonald criteria, or if they were found to have any MS diagnostic codes in their records. Application of these inclusion and exclusion criteria resulted in a dataset of 128,576 participants, including 9662 people with MS (7.5%) and 118,914 controls (92.5%; Fig. 1).

Of the 310,409 patients supplied by CPRD, the vast majority were registered at GP practices in England (309,657, 99.8%), with a small number located in Northern Ireland (752, 0.2%). Of the primary analysis population, the majority of MS cases (9598/9662, 99.34%) and controls (118,649/118914, 99.78%) were from England—the remainder of the cohort were from Northern Ireland (cases: 64/9662, 0.66%; controls: 265/118914, 0.22%). As patient-level IMD data were only available for participants registered in England, analyses adjusting for deprivation status were conducted without the 329 Northern Irish participants.

### Demographic, risk factor, and exposure definitions

Ethnicity was defined using a composite of HES data and primary care codes for self- or clinical-reported ethnic background. We grouped ethnicity codes into ‘White’, ‘Black’, ‘Asian’, and ‘Mixed/Other’, corresponding to UK Census categories. Where necessary due to low case/exposure counts, we simplified ethnicity into a binary variable (‘White’ or ‘Diverse’), in which people with coded ‘Black’, ‘Asian’, or ‘Mixed/Other’ ethnicity were grouped together. The ‘Asian’ group was largely made up of persons of reported Indian, Bangladeshi, or Pakistani ethnicity, and so we use the term ‘South Asian’ to refer to this group.

We selected established or putative risk/protective factors for MS based on consensus from recent meta-analyses and systematic reviews of observational studies [4, 5, 46] and availability of exposure data or reasonable proxies in CPRD. We included the following risk/protective factors: high BMI during early adulthood (aged 16–25), smoking, vitamin D status, infectious mononucleosis (IM), head injury, and alcohol consumption. To mitigate bias from reverse causation (e.g. MS causing changes in smoking behaviour), we only considered exposures occurring more than five years prior to the index date. IM cases were defined using recorded diagnoses only, i.e. serological data were not included, mainly due to the sparsity of these data.

Smoking status, BMI, IM, vitamin D insufficiency, alcohol consumption, and head injury were defined using primary care codes (Supplementary Materials). BMI was either taken from directly recorded BMI values or calculated from height and weight (weight in Kg/[height in M] [2]). BMI was defined as the earliest valid BMI recording after age 16, before the age of 25, and at least five years prior to the index date. BMI categories were determined using the WHO cut-offs: healthy weight (18.5–25), underweight (< 18.5), overweight (25–30), obese (30–40), and morbidly obese (> 40). Smoking status was dichotomised as ever vs never-smoking for each individual using codes recording smoking behaviour (supplementary material). We classified individuals as smokers if they had a code indicating that they smoked at least five years prior to the index date. If an individual had no recordings indicating they smoked and they had a positive recording indicating they had never smoked, we classified them as never-smokers. Individuals with no smoking status recorded were coded as having missing smoking data.

### Statistical analysis

#### Validation of established modifiable risk factors for MS

To determine the association between previously established risk/protective factors and MS risk in the CPRD cohort, we used multivariable logistic regression models to examine the association between each MS risk factor and MS status adjusting for index age and gender. ‘Index age’ was defined as the age at recorded MS diagnosis (for cases), and the age at recorded MS diagnosis for the matched case (for controls). For these analyses we used data from the entire cohort following the application of inclusion and exclusion criteria (see above). We also performed sensitivity analyses adjusting for deprivation status (index age, gender, and IMD quintile) and for ethnicity (index age, gender, and UK Census ethnicity category). To determine whether risk factors exerted independent effects, we also constructed a multivariable model adjusting for index age, gender, and all six risk factors simultaneously (raised BMI, smoking, vitamin D deficiency, head injury, IM, and alcohol consumption). Statistical significance was established using a likelihood ratio test, comparing the full model to a null model consisting of only index age, age at registration, and gender.

#### Consistency of MS risk factors across ethnic backgrounds

To examine whether the effects of MS risk factors varied according to ethnic background, we used multivariable unconditional logistic regression with MS status as the outcome and each exposure as the independent variable. We first assessed whether an interaction term (ethnicity × exposure) improved the fit of the model compared to a null model with only the main effects included. We used likelihood ratio tests to compare model fit. As a complementary approach, we performed stratified analysis, modelling the effect of each exposure on MS risk within each ethnicity category separately. Models were adjusted for index age and gender.

We then performed sensitivity analyses adjusting for deprivation status (IMD quintile considered as a continuous variable) in addition to index age and gender. We also performed a further sensitivity analysis with a more stringent case definition, stipulating that MS cases had to have an MS diagnostic code in both primary care and HES data. MS cases without a HES code for MS were excluded from these models. For the HES-MS cohort, we only included controls which had been matched to an included case.

#### General statistical methods

All analyses were adjusted for multiple testing using the Bonferroni correction, to maintain an *α* of 0.05. Unless specified, counts are presented as *n* (% of those with non-missing data) and continuous variables are presented as mean (SD). Odds ratios are presented with the 95% confidence interval, and missing data were excluded (i.e. we performed complete-case analysis). We also confirmed the association between each risk factor and MS status in models accounting for missing data using inverse probability weighting (see supplementary data, section ‘Missing Data and Collider Bias’). Descriptive statistics are shown in the tables (t tests for normally distributed continuous variables and chi-squared tests for categorical variables). P values for model fit are likelihood ratio test P values.

## Results

### Variation in MS demographics by ethnicity

We included 9,662 multiple sclerosis (MS) cases and 118,914 controls enrolled in the UK CPRD Aurum primary care dataset in the primary analysis. Demographic characteristics of the controls were representative of the UK population [47–49] (Table 1). The MS cohort were younger than controls at GP registration (27.6 [SD 14.4] vs 31.7 [SD 15.8]) with a higher proportion of women (70.6% vs 50.6%, *p* < 0.0001), were from less deprived areas (23.0% vs 20.7% in the most affluent IMD quintile, *p* < 0.0001), and were more likely to identify as White (92.5% vs 85.5%, *p* < 0.0001).

**Table 1:** Demographic characteristics of MS cases and controls in the UK CPRD Aurum dataset

	MS status		UK Census (2011)
	Control	Case	
*N*	118,914	9662	56.1 million
Gender			
Female	60,130 (50.6%)	6826 (70.6%)	50.1%
Male	58,784 (49.4%)	2836 (29.4%)	49.2%
Year of birth	1963.8 (15.8)	1965.4 (13.7)	
Index age (years)	47.1 (13.9)	45.6 (12.3)	Median 39
Data prior to index date (years)	15.3 (9.9)	18 (11.2)	
Ethnic background			
White	75,860 (85.5%)	8783 (92.5%)	86%
Asian	5043 (5.7%)	277 (2.9%)	5.3%
Black	4019 (4.5%)	251 (2.6%)	3.4%
Mixed/Other	3835 (4.3%)	189 (2%)	5.3%
IMD quintile			
1 (least deprived)	23,352 (20.7%)	2171 (23%)	
2	23,024 (20.4%)	2111 (22.4%)	
3	22,510 (20%)	1906 (20.2%)	
4	23,196 (20.6%)	1740 (18.5%)	
5 (most deprived)	20,527 (18.2%)	1500 (15.9%)	
Location			
Rural	17,032 (15.1%)	1707 (18.1%)	
Urban	95,577 (84.9%)	7721 (81.9%)	

Both MS and control cohorts were ethnically diverse (Table 2): Of the 9662 people with MS, 277 were South Asian (2.9%), 251 were Black (2.6%); of the 118,914 controls, 5043 were South Asian (5.7%) and 4019 were Black (4.5%). The age at MS first diagnostic code report was earlier in the Black (40.5 [SD 10.9]) and Asian (37.2 [SD 10.0]) ethnic groups compared with the White cohort (46.1 [SD 12.2]). There was a female predominance in all ethnic groups; however, the relative proportion of males was higher in the South Asian cohort (proportion of women 60.3% vs 71% [White] and 75.7% [Black]).

**Table 2:** Demographic characteristics of MS cases and controls from White, Black, South Asian, Mixed/Other, and Unknown ethnic backgrounds

	Ethnicity					
	White	Asian	Black	Mixed/Other	Missing	*P* value
MS CASES (*N* = 9662)						
N	8783	277	251	189	162	
Gender						0.0002
Female	6238 (71%)	167 (60.3%)	190 (75.7%)	126 (66.7%)	105 (64.8%)	
Male	2545 (29%)	110 (39.7%)	61 (24.3%)	63 (33.3%)	57 (35.2%)	
Year of birth	1964.6 (13.5)	1976.3 (10.9)	1972.2 (12.2)	1974.8 (12.9)	1964.8 (14.6)	< 0.0001
Index age (years)	46.1 (12.2)	37.2 (10)	40.5 (10.9)	38.6 (12.1)	44.4 (12.4)	< 0.0001
Data prior to index date (years)	18.2 (11.4)	14.7 (8.3)	15.2 (8.5)	14.6 (8)	18.9 (10.6)	< 0.0001
IMD quintile						< 0.0001
1 (least deprived)	2080 (24.1%)	40 (14.7%)	7 (2.9%)	33 (17.6%)	11 (13.3%)	
2	1994 (23.1%)	47 (17.3%)	21 (8.6%)	26 (13.8%)	23 (27.7%)	
3	1766 (20.4%)	50 (18.4%)	46 (18.9%)	25 (13.3%)	19 (22.9%)	
4	1514 (17.5%)	68 (25%)	86 (35.4%)	57 (30.3%)	15 (18.1%)	
5 (most deprived)	1288 (14.9%)	67 (24.6%)	83 (34.2%)	47 (25%)	15 (18.1%)	
Location						< 0.0001
Rural	1671 (19.3%)	2 (0.7%)	1 (0.4%)	14 (7.4%)	19 (22.9%)	
Urban	6971 (80.7%)	270 (99.3%)	242 (99.6%)	174 (92.6%)	64 (77.1%)	
CONTROLS (*N* = 118,914)						
*N*	75,860	5043	4019	3835	30,157	
Gender						< 0.0001
Female	39,183 (51.7%)	2451 (48.6%)	2134 (53.1%)	2000 (52.2%)	14,362 (47.6%)	
Male	36,677 (48.3%)	2592 (51.4%)	1885 (46.9%)	1835 (47.8%)	15,795 (52.4%)	
Year of birth	1962.7 (16.3)	1968.8 (14.6)	1968.4 (13.9)	1970.7 (13.9)	1964.2 (14.5)	< 0.0001
Index age (years)	48.2 (14.3)	44.5 (12.7)	44.6 (12.3)	43 (12.1)	45.3 (13)	< 0.0001
Data prior to index date (years)	15.1 (10.2)	11.9 (6.8)	11.5 (6.3)	11.2 (6.3)	17.4 (9.7)	< 0.0001
IMD quintile						< 0.0001
1 (least deprived)	16,459 (22.1%)	533 (10.8%)	158 (4%)	491 (12.9%)	5711 (22.6%)	
2	16,146 (21.6%)	709 (14.4%)	282 (7.2%)	585 (15.4%)	5302 (20.9%)	
3	14,930 (20%)	1012 (20.5%)	676 (17.2%)	834 (22%)	5058 (20%)	
4	14,259 (19.1%)	1400 (28.3%)	1398 (35.6%)	996 (26.3%)	5143 (20.3%)	
5 (most deprived)	12,831 (17.2%)	1285 (26%)	1417 (36%)	887 (23.4%)	4107 (16.2%)	
Location						< 0.0001
Rural	13,161 (17.6%)	93 (1.9%)	56 (1.4%)	168 (4.4%)	3554 (14%)	
Urban	61,464 (82.4%)	4846 (98.1%)	3875 (98.6%)	3625 (95.6%)	21,767 (86%)	

### Validation of established modifiable risk factors for MS

**Fig. 2:**
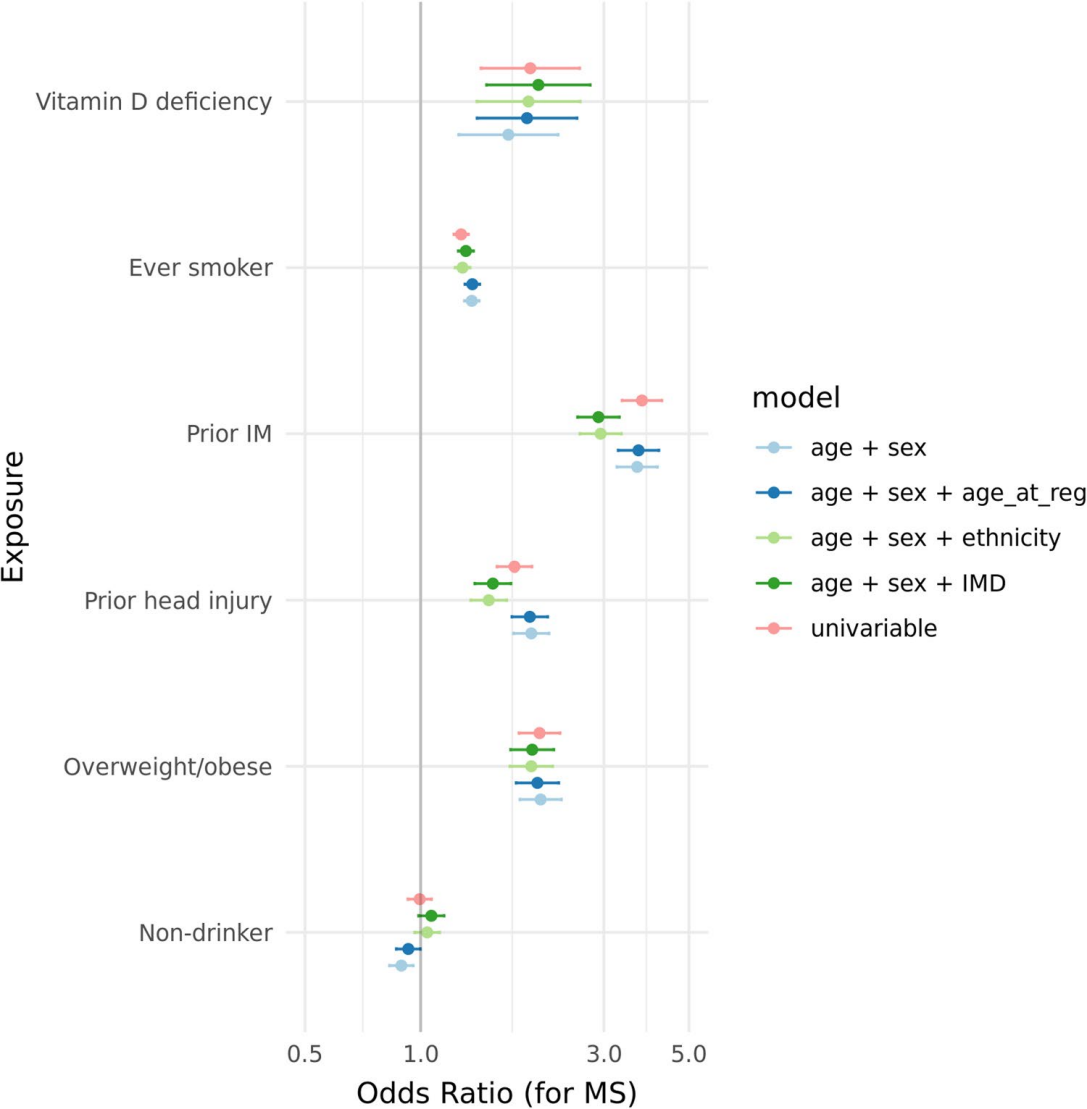
Case–control study of multiple sclerosis risk in CPRD Aurum recapitulates the roles of established exposures. Each point represents the odds ratio and its 95% confidence interval calculated from multivariable logistic regression models for each exposure, adjusted for age and gender. The y-axis indicates each exposure. The x-axis is on the log10 scale. Points are coloured according to which model they were derived from. The primary analysis models adjusted for index age and gender. Secondary sensitivity analyses adjusted for age at registration (age_at_reg), ethnicity, and deprivation status (IMD; Indices of Multiple Deprivation)

To ensure that the epidemiological characteristics of MS in this cohort mirrored those of previously described cohorts, we first sought to validate the effects of established modifiable MS risk factors across the entire cohort (Table 3). Consistent with previous studies, we observed associations (*P*_adjusted_ < 0.05) between risk of MS and higher BMI (OR 2.05, 95% CI 1.81–2.33 for overweight/obesity), current or previous smoking (OR 1.36, 95% CI 1.30–1.42), infectious mononucleosis (IM; OR 3.66, 95% CI 3.25–4.14), vitamin D deficiency/insufficiency (OR 1.69, 95% CI 1.26–2.28), and head injury (OR 1.94, 95% CI 1.75–2.16) (Table 3, Fig. 2).

**Table 3:** Results from multivariable logistic regression models examining the effect of selected exposures on subsequent MS risk

	*N*				Model							
	Controls		MS		Age + Sex (primary)		Ethnicity		Deprivation		Multivariable	
Risk factor	Unexp	Exp	Unexp	Exp	OR (95% CI)	*P*	OR (95% CI)	*P*	OR (95% CI)	*P*	OR (95% CI)	*P*
Raised BMI	7812	2478	751	486	2.05 (1.81–2.33)	< 0.0001	1.94 (1.71–2.21)	< 0.0001	1.95 (1.72–2.22)	< 0.0001	1.68 (1.5–1.89)	< 0.0001
Smoking	44,332	47,546	3862	5282	1.36 (1.3–1.42)	< 0.0001	1.29 (1.23–1.34)	< 0.0001	1.31 (1.25–1.37)	< 0.0001	1.97 (1.65–2.34)	< 0.0001
IM	117,699	1215	9300	362	3.66 (3.25–4.14)	< 0.0001	2.94 (2.6–3.33)	< 0.0001	2.9 (2.56–3.29)	< 0.0001	3.28 (2.27–4.75)	< 0.0001
Vitamin D deficiency	118,543	371	9605	57	1.69 (1.26–2.28)	0.001	1.91 (1.4–2.6)	< 0.0001	2.03 (1.49–2.76)	< 0.0001	3.1 (1.38–6.98)	0.006
Alcohol abstinence	42,062	11,603	3934	1079	0.89 (0.83–0.96)	0.001	1.04 (0.97–1.12)	0.303	1.07 (0.99–1.15)	0.099	1.16 (0.96–1.4)	0.11
Head injury	115,943	2971	9246	416	1.94 (1.75–2.16)	< 0.0001	1.5 (1.35–1.68)	< 0.0001	1.54 (1.38–1.72)	< 0.0001	2.36 (1.69–3.3)	< 0.0001

For each exposure, the *N* indicates the number of individuals with non-missing data used in the model (divided according to exposed vs non-exposed status [‘Exp’ and ‘Unexp’, respectively] and case–control status)

For each model, the Odds ratio for MS, 95% CI, and Wald test *P* value are reported. We report results from the primary analysis model (adjusted for index age and gender), and sensitivity analyses adjusting for ethnicity (in addition to index age and gender) and IMD quintile (in addition to index age and gender)

We also observed weak evidence for an association between alcohol consumption and MS (OR for non-drinkers 0.89, 95% CI 0.83–0.96); this effect was inconsistent across sensitivity analyses and dissipated on adjustment for ethnicity and deprivation status, suggesting that this effect is likely a result of confounding rather than an independent risk factor (Table 3). In a combined model examining the impact of all six risk factors jointly, we observed independent effects of raised BMI, IM, vitamin D deficiency, smoking, and head injury on MS risk, whereas the impact of alcohol consumption was diminished (Table 3).

All risk factors except alcohol consumption were associated with MS in sensitivity analyses adjusting for ethnicity or deprivation status (Fig. 2). Furthermore, we obtained similar results in sensitivity analyses restricting to cases with HES-confirmed MS (*N*_MS_ = 6870, *N*_Control_ = 40,982). We observed the expected dose–response relationships between early adulthood BMI and MS risk, with higher levels of exposure conferring higher risk of MS. The impact of obesity (OR 2.7, 95% CI 2.2–3.2; *N*_MS_ = 166, *N*_Control_ = 666) or morbid obesity (OR 4.2, 95% CI 2.8–6.4; *N*_MS_ = 32, *N*_Control_ = 81) exceeded that of overweight (OR 1.8, 95% CI 1.5–2.1; *N*_MS_ = 288, *N*_Control_ = 1731).

### Consistency of MS risk factors across ethnic backgrounds

**Fig. 3:**
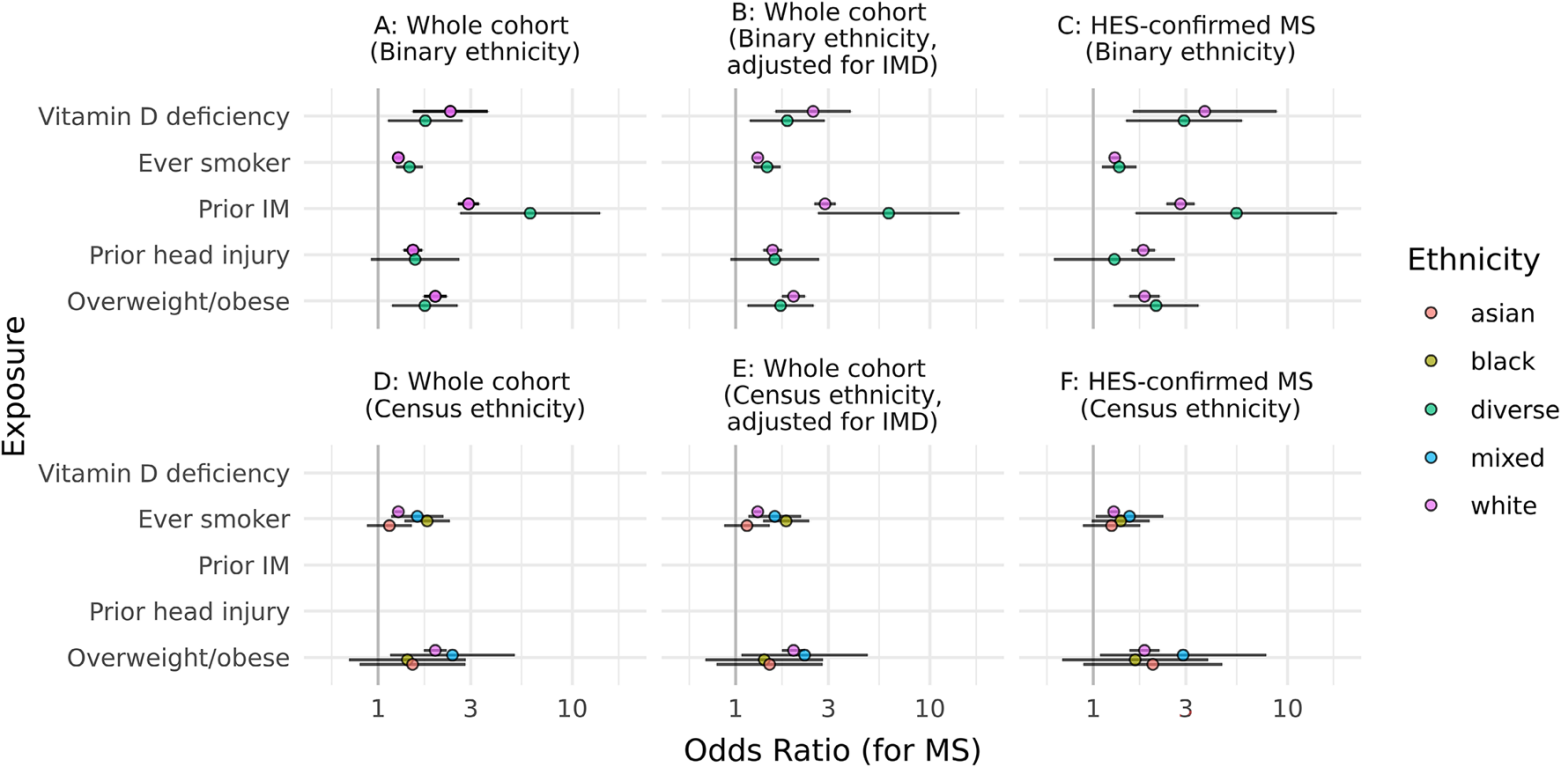
Stratified analysis of MS risk factors according to ethnic background. Odds ratios and 95% confidence intervals for multiple sclerosis given each exposure on the y axis. Odds ratios represent the estimates from logistic regression models adjusted for index age and gender. Effect estimates are coloured according to ethnic background. Several forest plots are presented, representing different sensitivity analyses. **A** Analysis of the whole cohort is shown, with ethnicity dichotomised into ‘White’ and ‘Diverse’ ethnic groups. **B** Analysis of the whole cohort, models adjusted for index age, gender, and deprivation status (i.e. IMD quintile). **C** cohort restricted to cases with HES-confirmed MS, i.e. orthogonal evidence of having MS provided by hospital episode statistics. Models adjusted for index age and gender. **E**, **F** Analyses depict models stratified by more granular ethnicity categories, corresponding to UK Census categories. Only BMI and smoking were analysed in this way due to low case numbers for other exposures. **D** Analysis of the whole cohort, models adjusted for index age and gender. **E** Whole cohort, models adjusted for index age, gender, and deprivation status. **F** HES-confirmed MS, models adjusted for index age and gender. The x-axis is on a log10 scale

Having validated the association of established MS risk factors in the entire case–control cohort, we next considered whether their effect was modified by ethnic background. Although the cohort is diverse (*N*_MS_: 277 South Asian, 251 Black, 8783 White; *N*_Control_: 5043 South Asian, 4019 Black, 75,860 White), the numbers of cases from South Asian or Black backgrounds with coded IM, vitamin D deficiency, or head injury was low (Table 4). To circumvent issues with model stability, we therefore dichotomised ethnic background into ‘White’ and ‘South Asian/Black/Mixed/Other’ (termed ‘Diverse’). We found evidence for directionally consistent effects of all tested exposures between the ‘White’ and ‘Diverse’ ethnic groups (Table 4; Fig. 3).

**Table 4:** Results from multivariable logistic regression models examining the effect of selected exposures on subsequent MS risk stratified by ethnic background

		Controls		MS				
Risk factor	Ethnicity	Unexp	Exp	Unexp	Exp	OR (95% CI)	*P*	*P*_int_
Raised BMI	White	4848	1634	655	432	1.97 (1.72–2.26)	< 0.0001	0.676
	Asian	364	110	38	17	1.5 (0.8–2.82)	0.202	
	Black	199	100	22	16	1.42 (0.71–2.83)	0.325	
	Mixed/Other	323	89	22	13	2.42 (1.15–5.06)	0.019	
Smoking	White	29,815	35,694	3406	4909	1.27 (1.21–1.33)	< 0.0001	0.102
	Asian	2803	1586	175	95	1.14 (0.88–1.49)	0.322	
	Black	2066	1358	121	118	1.79 (1.37–2.34)	< 0.0001	
	Mixed/Other	1800	1517	83	93	1.59 (1.17–2.17)	0.003	
IM	White	74,844	1016	8431	352	2.92 (2.58–3.31)	< 0.0001	0.083
	Asian	5037	6	277	< 5	6.07 (2.65–13.9)	< 0.0001	
	Black	4012	7	248	< 5			
	Mixed/Other	3826	9	184	5			
Vitamin D deficiency	White	75,773	87	8757	26	2.36 (1.51–3.67)	< 0.0001	0.23
	Asian	4901	142	263	14	1.75 (1.12–2.72)	0.013	
	Black	3961	58	244	7			
	Mixed/Other	3800	35	187	< 5			
Head injury	White	73,364	2496	8386	397	1.51 (1.35–1.69)	< 0.0001	0.608
	Asian	4970	73	272	5	1.55 (0.92–2.62)	0.101	
	Black	3965	54	244	7			
	Mixed/Other	3777	58	185	< 5			

For each exposure, the *N* indicates the number of individuals with non-missing data used in the model (divided according to exposed vs non-exposed status [‘Exp’ and ‘Unexp’, respectively], case–control status, and ethnic background). For each model, the Odds ratio for MS, 95% CI, and Wald test *P* value are reported. Odds ratios and 95% confidence intervals represent the output of logistic regression models stratified by ethnicity, adjusted for index age, and gender. Note that for overweight/obesity and smoking, we stratified by Census categories of ethnic background (i.e. White, South Asian, Black, and Mixed/Other), whereas for the other risk factors, we simplified ethnicity into a binary ‘White’/’Diverse’ variable due to the small numbers of cases

*P* values for interaction (*P*_int_) are reported, representing the improvement in model fit afforded by inclusion of a ‘risk factor x ethnicity’ interaction term. As per CPRD reporting policy, cells with fewer than five events are reported as < 5

There was no evidence of statistical interaction between ethnicity—dichotomised as ‘White’ vs ‘Diverse’—and any of the following risk factors: elevated BMI prior to age 25 (White: OR 1.97, 95% CI 1.72–2.26, Diverse: OR 1.74, 95% CI 1.18–2.57, *P*_Interaction_ = 0.58), smoking (White: OR 1.27, 95% CI 1.21–1.33, Diverse: OR 1.45, 95% CI 1.24–1.70, *P*_Interaction_ = 0.31), prior IM (White: OR 2.92, 95% CI 2.58–3.31, Diverse: OR 6.07, 95% CI 2.65–13.90, *P*_Interaction_ = 0.08), vitamin D deficiency (White: OR 2.36, 95% CI 1.51–3.67, Diverse: OR 1.75, 95% CI 1.12–2.71, *P*_Interaction_ = 0.23), or head injury (White: OR 1.51, 95% CI 1.35–1.69, Diverse: OR 1.55, 95% CI 0.92–2.62, *P*_Interaction_ = 0.61).

We repeated these analyses with a more refined definition of ethnicity where there were sufficient numbers of cases exposed to the risk factor in question (i.e. greater than ten events in each group [50]). Due to small numbers of Black and South Asian participants with MS exposed to prior IM, head injury, or vitamin D deficiency, we analysed the impact of obesity and smoking across ethnic groups. Broadly speaking, these results demonstrated consistent effects of smoking and obesity on MS risk across ethnic groups with no evidence of statistical interaction between ethnicity and either risk factor (Fig. 3 and Table 4).

The impact of obesity appeared consistent across ethnic groups (White: OR 1.97, 95% CI 1.72–2.26; Asian: OR 1.50, 95% CI 0.80–2.82; Black: OR 1.42, 95% CI 0.71–2.83; *P*_Interaction_ = 0.68). We observed a similar result when considering the impact of BMI as a continuous variable (White: OR 1.30, 95% CI 1.23–1.38, Asian: OR 1.10, 95% CI 0.86–1.41; Black: OR 1.20, 95% CI 0.94–1.53; *P*_Interaction_ = 0.06). Prior smoking also appeared to influence MS risk in a consistent manner across ethnic groups (White: OR 1.27, 95% CI 1.21–1.33; Asian: OR 1.14, 95% CI 0.88–1.49; Black: OR 1.79, 95% CI 1.37–2.34; *P*_Interaction_ = 0.10). Due to the relatively small sample sizes, the confidence intervals for effect estimates in the Black and South Asian groups were broad, but importantly the effect estimates are all in the same direction, suggesting that raised BMI and smoking act as risk factors across ethnic groups.

Deprivation could plausibly act as a confounder, both due to its associations with established risk factors (e.g. smoking behaviour) and due to differential access to healthcare services. We performed sensitivity analyses adjusting for deprivation (quantified by the indices of multiple deprivation [IMD] quintile) in addition to index age and gender. These models yielded similar results to the main analysis, with consistent effect estimates for all risk factors between ‘White’ and ‘Diverse’ ethnic groups and no strong statistical evidence of interaction between any risk factor and ethnicity (Fig. 3). We obtained similar results using a more stringent case definition (i.e. restricting to MS cases with a HES diagnostic code; *N*_MS_ = 6870) (Fig. 3).

## Discussion

In this study, we use data from CPRD—a population-based UK cohort—to determine whether potentially modifiable risk factors for multiple sclerosis have distinct effects across ethnic backgrounds and strata of deprivation in England. These analyses demonstrated that modifiable risk factors for MS previously reported in White populations—smoking, obesity, head injury, infectious mononucleosis, and vitamin D deficiency—are also likely risk factors for MS across South Asian and Black ethnic backgrounds.

We provide the clearest evidence to date that the established modifiable risk factors for MS—smoking, obesity, infectious mononucleosis, vitamin D deficiency, and head injury—have similar implications for subsequent MS risk, regardless of demographic background. We find that the effects of these risk factors are consistent—in terms of direction—across ethnic groups, with no statistical evidence for an interaction between any exposure and ethnicity. The lack of statistical interaction on the multiplicative scale argues for a broadly similar impact of these risk factors across ethnic groups; however, we cannot definitely claim that the magnitude of these effects is identical due to the small numbers of cases exposed to some risk factors (e.g. IM) and the lack of truly population-based data (this is a nested case-control study within a population cohort), which are required to assess the absolute risk difference conferred by exposure to the risk factors under study.

These results increase confidence that efforts to reduce the population incidence of MS by targeting these exposures should have potential benefit for all ethnic groups. We also report an earlier age of onset in Black and Asian individuals with MS [6, 51], consistent with previous findings, and a weaker female predominance in Asian individuals, which is a novel finding to the best of our knowledge [7, 51].

Relatively few studies have examined the role of MS risk factors across ethnic groups, at least in part due to the size and diversity of the cohort required. Another UK population-based electronic healthcare record (EHR) study reported that the effects of smoking and IM on MS risk may be greater among Black individuals—while the biological interpretation of this statistical interaction is unclear, a key observation is that the effects of IM and smoking were concordant in direction in across ethnic groups [3]. A US cohort study found that there was a lack of evidence for association between low serum vitamin D and MS risk in Black and Hispanic American individuals, but a consistent relationship with lifetime sun exposure [18]. In the same cohort, a consistent relationship between EBV (EBNA-1) seropositivity and MS has been reported across ethnicities, in contrast to the inconsistent relationship with CMV seropositivity [19]. Our findings reinforce the view supported by previous data that in general, modifiable risk factors for MS which have been validated in White European/American cohorts are also risk factors among other ethnic groups.

It is important to note that although some of the statistical tests for multiplicative interaction were weakly suggestive of a quantitative interaction, with the effect of the exposure differing in magnitude but not direction, these statistical effects are not likely to be biologically relevant. None of the risk factors examined show evidence of qualitative interaction, i.e. a reversal of effect or an absence of effect in one group [52]. Some estimates in the ethnicity-stratified models are imprecise due to small numbers, and so although the confidence interval crosses the null this is perhaps best interpreted as the absence of evidence for heterogeneity of effects rather than evidence of the absence of an effect.

There are some important limitations to this study. First, we report findings from a single dataset without external replication. Although we had hoped to replicate our findings in CPRD GOLD, the companion dataset to CPRD Aurum, the numbers of individuals with MS from Asian (*n* = 50) and Black (*n* = 43) backgrounds was too low to allow for meaningful analysis. External replication in a separate dataset is required to increase the confidence in our findings—drives to improve diversity in MS cohorts are essential to ensure this question and similar questions can be addressed in the future.

Second, as data are routinely recorded, there are many missing data points, both for important covariates such as ethnicity and for exposures such as BMI. For instance, the prevalence of recorded vitamin D deficiency in the MS cohort is almost tenfold lower than published estimates (~ 5% in our study vs over 50% in the BENEFIT trial [53])—this is likely to reflect under-ascertainment, with the majority of cases of asymptomatic deficiency/insufficiency remaining unrecorded. Missing data and under-ascertainment are inescapable consequences of using electronic healthcare record data, limit our power for all exposures except those routinely recorded in primary care—BMI and smoking—and could introduce bias. Non-random missingness may introduce collider bias, which could distort our findings in either direction. By restricting our analyses to participants with an index date of 2001 or later, we minimise the risk that non-random missingness for ethnicity data could distort our findings as ethnicity recording has improved substantially in CPRD from around this time [54]. Furthermore, the population characteristics of the control cohort closely resemble those of the UK census population, and the MS cohort mirrors previously described MS cohorts. These factors argue against non-random missingness being a major source of bias in this study.

Third, the definition of the outcome—MS—is derived from electronic healthcare records and so is likely to be less specific than criteria-defined MS diagnosed by a neurologist. Nevertheless, our use of two or more diagnostic codes, triangulation with HES data, exclusion of several diagnostic codes for conditions which could mimic MS, and restricting to participants with an index date after the initial publication of the McDonald diagnostic criteria should increase the accuracy of our outcome definition. Chronic conditions such as MS are also likely to be ‘back-coded’ by primary care practitioners following diagnosis in secondary care. This dataset has also been used by several other groups to examine aspects of MS epidemiology [40, 42, 44, 49] and recapitulates the role of several established modifiable risk factors. The exposure definitions are also derived from EHR codes, and are therefore by necessity simplifications of real-world exposure to risk factors. For instance, we use the earliest BMI recording between the ages of 16 and 25 as a proxy for the established MS risk factor, obesity during adolescence. This measure does not capture fluctuations in BMI, inaccuracies in the recording of BMI, or the fact that BMI is an imperfect measure of adiposity which may be particularly inaccurate in people from certain ethnic backgrounds [55].

Fourth, due to the relatively small numbers of cases exposed to certain risk factors in the Black or South Asian ethnic groups, we were unable to meaningfully report on stratified regression models examining the impact of these risk factors separately in each ethnic group. We collapsed these groups into a single category—‘diverse’—to allow for statistical comparison with the effect of risk factors in participants identifying as White. While this approach was successful in allowing us to demonstrate consistency of these risk factors regardless of ethnicity, it is a significant simplification and should be interpreted as such. Ideally, these analyses should be replicated in cohorts with even greater sample sizes so that more granular analysis can be performed.

There are also some key strengths of this cohort and our study design. The diversity of the CPRD cohort, with over 200 MS cases in the South Asian and Black ethnic groups, makes it a valuable resource for drawing inferences about the causes of MS across diverse backgrounds. The size of this cohort and the wealth of data available for each participant allow us to systematically examine the effects of multiple exposures on MS risk while controlling for relevant confounders within ethnic groups—our sample sizes within each ethnic group surpass those of previous studies. The magnitude of effects we observe for the association between modifiable exposures and MS is broadly consistent with previous studies. We do not see evidence for an association with alcohol consumption, in contrast to some previous reports but consistent with our previous finding in UK Biobank [56]. The population-based design of CPRD reduces the risk of selection bias, and the large size of the sample permits statistical tests for interaction.

In summary, using a large primary care dataset covering >10% of the UK population, we provide the strongest evidence to date that modifiable risk factors for multiple sclerosis previously validated in people of White ethnic backgrounds are of similar relevance for persons of South Asian or Black ethnicity. These findings will have implications for prevention efforts targeting these risk factors.

## Data Availability

All analyses were conducted in R version 4.1.1 via the Queen Mary University of London Apocrita High-Performance Computing (HPC) facility. All code is available at https://github.com/benjacobs123456/cprd including diagnostic code lists used for exposure definitions.

## Abbreviations

AE: Accident and emergency
APC: Admitted patient care
CNS: Central nervous system
CPRD: Clinical practice research datalink
GP: General practice
HES: Hospital episode statistics
IM: Infectious mononucleosis
IMD: Index of multiple deprivation
LSOA: Lower layer super output area
MR: Mendelian randomisation
MS: Multiple sclerosis
ONS: Office of National Statistics
OP: Outpatients
UK: United Kingdom

## Data availability

Data are available from CPRD on request—details are available at https://cprd.com/data-access.

## Declarations

### Reporting guidelines

This research was conducted and reported in accordance with the STROBE guidelines on observational studies. 
